# Generation of SNP datasets for orangutan population genomics using improved reduced-representation sequencing and direct comparisons of SNP calling algorithms

**DOI:** 10.1186/1471-2164-15-16

**Published:** 2014-01-10

**Authors:** Maja P Greminger, Kai N Stölting, Alexander Nater, Benoit Goossens, Natasha Arora, Rémy Bruggmann, Andrea Patrignani, Beatrice Nussberger, Reeta Sharma, Robert H S Kraus, Laurentius N Ambu, Ian Singleton, Lounes Chikhi, Carel P van Schaik, Michael Krützen

**Affiliations:** 1 Evolutionary Genetics Group, Anthropological Institute and Museum, University of Zurich, Zurich, Switzerland; 2 Unit of Ecology & Evolution, Department of Biology, University of Fribourg, Fribourg, Switzerland; 3 Organisms and Environment Division, School of Biosciences, Cardiff University, Cardiff, UK; 4 Danau Girang Field Centre, c/o Sabah Wildlife Department, Kota Kinabalu, Sabah, Malaysia; 5 Sabah Wildlife Department, Kota Kinabalu, Sabah, Malaysia; 6 Functional Genomics Center, University of Zurich, Zurich, Switzerland; 7 Department of Biology, University of Berne, Berne, Switzerland; 8 Institute of Evolutionary Biology and Environmental Studies, University of Zurich, Zurich, Switzerland; 9 Population and Conservation Genetics, Instituto Gulbenkian de Ciencia, Oeiras, Portugal; 10 Conservation Genetics Group, Senckenberg Research Institute and Natural History Museum, Gelnhausen, Germany; 11 Foundation for a Sustainable Ecosystem (YEL), Medan, Indonesia; 12 PanEco, Foundation for Sustainable Development and Intercultural Exchange, Berg am Irchel, Switzerland; 13 CNRS, Laboratoire Evolution and Diversité Biologique, Université Paul Sabatier, Toulouse, France; 14 Université de Toulouse, Toulouse, France

**Keywords:** Next-generation sequencing, Single-nucleotide polymorphisms, Reduced-representation libraries, Bioinformatics, GATK, SAMtools, CLC genomics workbench, Great apes

## Abstract

**Background:**

High-throughput sequencing has opened up exciting possibilities in population and conservation genetics by enabling the assessment of genetic variation at genome-wide scales. One approach to reduce genome complexity, i.e. investigating only parts of the genome, is reduced-representation library (RRL) sequencing. Like similar approaches, RRL sequencing reduces ascertainment bias due to simultaneous discovery and genotyping of single-nucleotide polymorphisms (SNPs) and does not require reference genomes. Yet, generating such datasets remains challenging due to laboratory and bioinformatical issues. In the laboratory, current protocols require improvements with regards to sequencing homologous fragments to reduce the number of missing genotypes. From the bioinformatical perspective, the reliance of most studies on a single SNP caller disregards the possibility that different algorithms may produce disparate SNP datasets.

**Results:**

We present an improved RRL (iRRL) protocol that maximizes the generation of homologous DNA sequences, thus achieving improved genotyping-by-sequencing efficiency. Our modifications facilitate generation of single-sample libraries, enabling individual genotype assignments instead of pooled-sample analysis. We sequenced ~1% of the orangutan genome with 41-fold median coverage in 31 wild-born individuals from two populations. SNPs and genotypes were called using three different algorithms. We obtained substantially different SNP datasets depending on the SNP caller. Genotype validations revealed that the *Unified Genotyper* of the *Genome Analysis Toolkit* and *SAMtools* performed significantly better than a caller from *CLC Genomics Workbench* (CLC). Of all conflicting genotype calls, CLC was only correct in 17% of the cases. Furthermore, conflicting genotypes between two algorithms showed a systematic bias in that one caller almost exclusively assigned heterozygotes, while the other one almost exclusively assigned homozygotes.

**Conclusions:**

Our enhanced iRRL approach greatly facilitates genotyping-by-sequencing and thus direct estimates of allele frequencies. Our direct comparison of three commonly used SNP callers emphasizes the need to question the accuracy of SNP and genotype calling, as we obtained considerably different SNP datasets depending on caller algorithms, sequencing depths and filtering criteria. These differences affected scans for signatures of natural selection, but will also exert undue influences on demographic inferences. This study presents the first effort to generate a population genomic dataset for wild-born orangutans with known population provenance.

## Background

The availability of high-throughput sequencing has revolutionized the fields of population genetics and molecular ecology [1]. Early genomic work focused mainly on broad comparative analyses between species [2-6] and was limited to one or a few individuals per species. The emergent field of population genomics [7], including conservation [8] and landscape genomics [9], investigates genomic allele-frequency patterns at the species level, i.e. among and within natural populations. Main interests revolve around exploring patterns of genetic diversity, differentiation and admixture, inferring demographic population histories, and studying signals of local adaptations in wild populations [7-9].

To date, population-genomics studies have mainly focused on humans [10,11], some model species [12-14] and others relevant to agricultural production [15,16]. Other taxa, particularly those with large genomes, have remained largely unexplored because of significant challenges in the laboratory and during bioinformatical analyses [8,17,18]. Sequencing of complete genomes of many individuals is usually still prohibitive because of associated costs and bioinformatical complexities, especially in species where a reference genome is unavailable. Yet, many biological questions can be addressed by describing polymorphisms from a subset of genomic regions, provided that these regions are approximately evenly distributed throughout the genome.

In the laboratory, several strategies have recently been developed enabling so-called ‘reduced genome complexity sequencing’, i.e. sampling only a small fraction of the genome in several individuals. These strategies include sequencing of reduced-representation libraries (RRLs) [19], restriction-site-associated DNA sequencing [7,20], and other sequence-based-genotyping approaches [21,22]. Essentially, all of these methods are based on the same key principle: reducing genome complexity by digestion of genomic DNA with one or several restriction enzymes followed by a selection of resulting restriction fragments, and high-throughput sequencing of the final set of fragments.

One of the key characteristics of the aforementioned methods is that, at least in theory, read mapping can be carried out regardless of the availability of a reference genome by constructing a reference sequence from overlapping sequence stacks [21,23-26]. Moreover, the similarity among sequence stacks of different individuals allows the direct estimation of allele frequencies by simultaneous identification of polymorphisms and genotype calling (genotyping-by-sequencing). This reduces the major issue of ascertainment bias, which arises when markers are identified in a small subset of individuals and subsequently genotyped in an extended sample set [1,17,27].

One popular reduced-genome complexity approach is RRL sequencing. RRLs were first used to generate single-nucleotide polymorphisms (SNP) maps of the human genome using classical Sanger sequencing [28]. Since Van Tassel *et al.*[19] first adapted the approach to high-throughput sequencing, it has been applied in a number of SNP discovery studies (e.g. [26,29-32]). In the RRL approach, the number of restriction fragments subjected to high-throughput sequencing is reduced via size-selection before sequencing library preparation. RRLs allow the degree of complexity reduction to be customized by defining the selected fragment-size range. By providing easy access to flanking sequences necessary to design SNP genotyping assays when a reference genome is unavailable, RRLs are superior to other reduced-complexity approaches [7,20]. In the RRL approach, long DNA stretches can be sequenced by simply size-selecting for longer fragments (up to several kb possible) and complete sequencing of these fragments independent of the platform read length through shearing of fragments prior to high-throughput sequencing library preparation followed by assembly of the resulting sequence fragments [30].

Although the RRL principle is highly promising for generating population genomic SNP data, current protocols must be improved so as to i) facilitate library construction for individual samples, and most importantly, ii) maximize the number of homologous fragments generated during library construction. In the past, RRL sequencing has usually been performed on pools of DNA samples from multiple individuals for practical reasons [23,26,32,33]. However, pooling leads to the loss of major biological information as it prohibits the assignment of individual genotypes (i.e. genotyping-by-sequencing). Because of this, many biological questions, such as investigating admixture or linking phenotypes with genotypes in studies of natural selection, cannot be addressed when samples are pooled. Furthermore, pooling strongly increases the risk of missing rare alleles, especially if there are many individuals in the pool [34]. In addition, pooling is highly sensitive to variation in DNA concentration among samples, which will inadvertently lead to an over – or underrepresentation of certain alleles [34]. Thus, current protocols need to be improved to facilitate RRL generation of individual samples.

Analyzing individual samples requires improvements to minimize DNA loss during purification steps, which is particularly important if sample-DNA quantity is limited. Moreover, genome complexity needs to be reduced in a reproducible manner (i.e. homologous sites must be sequenced) across samples as this primarily determines the effectiveness of the genotyping-by-sequencing principle and reference-free mapping [22]. Non-overlapping sequences will lead to a high number of missing genotypes. The accurate sequencing of homologous sites is also of particular importance when working with pooled samples, as the true number of sequenced individuals at a particular SNP site cannot be determined. In the most extreme case, only alleles of one individual would be sequenced. In such a case, however, allele frequencies would nonetheless be estimated under the assumption that all allele copies in the pool had been sampled.

From a bioinformatical point of view, the amounts of raw data produced by high-throughput sequencing platforms are vast and many computational steps are required to translate raw outputs into high-quality SNP calls [18]. Thus, accurately identifying SNPs and calling genotypes from high-throughput sequencing data while filtering out sequencing errors remains a challenge. Various SNP calling programs have been introduced and algorithms are under constant development [18,35-37].

One of the most widely used commercial software suites for genomic data analysis is the *CLC Genomics Workbench* (CLC bio, Aarhus, Denmark). The software contains a basic SNP caller (hereafter referred to as ‘CLC’) that detects SNPs based solely on applying quality thresholds to sequencing, mapping and base quality. Genotypes are determined using hard-filter criteria, i.e. by simply counting the number of sequencing reads for each allele and applying arbitrary custom cut-off rules. For instance, a genotype would be called heterozygous if an alternative allele is present in 20-80% of the reads. However, for low sequencing depths this way of genotype calling tends to underestimate the number of heterozygous genotypes [18].

Arguably, two of the most popular non-commercial software suites are the *Genome Analysis Toolkit* (Broad Institute) [37,38] and *SAMtools*[36]. Both *SAMtools* and the *Unified Genotyper* of the *Genome Analysis Toolkit* (hereafter referred to as ‘GATK’), incorporate uncertainty in a probabilistic framework, in order to call SNPs and genotypes simultaneously [36-38]. Both SAMtools and GATK allow the joint analysis of all samples from one population (multi-sample calling). A major strength of the Bayesian framework is the potential to incorporate prior information, such as previous observations of alternative alleles, heterozygosity, and allele frequencies. Ideally, additional information such as representative reference SNPs or linkage-disequilibrium patterns could be incorporated [18,37,39]. Unfortunately, such information so far limited to a few model species (e.g. Arabidopsis [12]) and humans [10]. It has been proposed that in contrast to CLC, GATK (and potentially also SAMtools) might have the tendency to overestimate the number of heterozygous genotypes [37]. This is because GATK aggressively calls alternative alleles in favor of high sensitivity, resulting in a high number of false-positive calls which require extensive post-filtering.

Despite the fact that accurate SNP and genotype calling is fundamental for precise population parameter estimation in downstream analyses [17,40,41], to our knowledge direct comparisons of different SNP callers in the aforementioned context are still scarce. To date, most studies employ only one SNP caller, although it is conceivable that different callers will produce different datasets. In previous studies, validations were often restricted to confirming and comparing the polymorphic state of SNPs (e.g. [26,31,42]), but not actual genotypes at the individual level.

Here, we provide a comprehensive framework to obtain high-quality SNP data in population genomics, addressing both laboratory and bioinformatic challenges. First, we refined and improved an RRL protocol (iRRL), which maximizes the generation of homologous DNA fragments across individuals, thus achieving high genotyping-by-sequencing efficiency. Our protocol also contains modifications for economical handling of DNA during library preparation. All modifications support the establishment of single-sample libraries. Second, we directly compared three popular SNP callers (GATK, SAMtools and CLC) using our iRRL data generated for two orangutan populations (Genus: *Pongo*).

Orangutans are the only great apes found outside Africa and the phylogenetically most distant great apes to humans, which makes them particularly interesting to study in terms of the evolution of the hominid lineage [43,44]. In contrast to humans (e.g. the *International HapMap Project*[10]; the *1000 Genomes Project*[11]), in non-human great apes large-scale population genomic data from wild-born individuals with known population origin are scarce (but see [45,46]). Rather, most genomic data were generated from a small number of zoo animals with mostly unknown population origins [47-50], thus providing a limited perspective for population genomic analyses of wild populations. Genome-wide data in orangutans will enable the investigation of the genetic basis of local adaptations among orangutan populations [51]. Moreover, population genomic data will shed more light on the particularly complex demographic history of orangutans, as shaped by volcanic eruptions and recurrent sea level changes connecting the islands of Borneo and Sumatra during the Pleistocene [52-56].

## Results

### Improved reduced-representation sequencing

We developed a protocol to construct improved RRLs (referred to as iRRLs) that maximizes efficiency and repeatability of genome complexity reduction. We applied several key modifications to the method outlined in van Tassel *et al.*[19] including: (i) high-resolution fragment-size-selection down to an accuracy of one base pair to increase precision of isolating homologous fragments (Additional file 1: Figure S2), (ii) modifications to minimize DNA loss during purification steps, achieving DNA recovery rates of >95%, and (iii) adjustments to establish single-sample libraries to avoid the necessity of sample pooling. In order for restriction enzymes to generate homologous fragments across samples, our protocol includes recommendations for suitable sample handling and DNA isolation to avoid DNA strand breaking prior to digestion.

**Figure 1:**
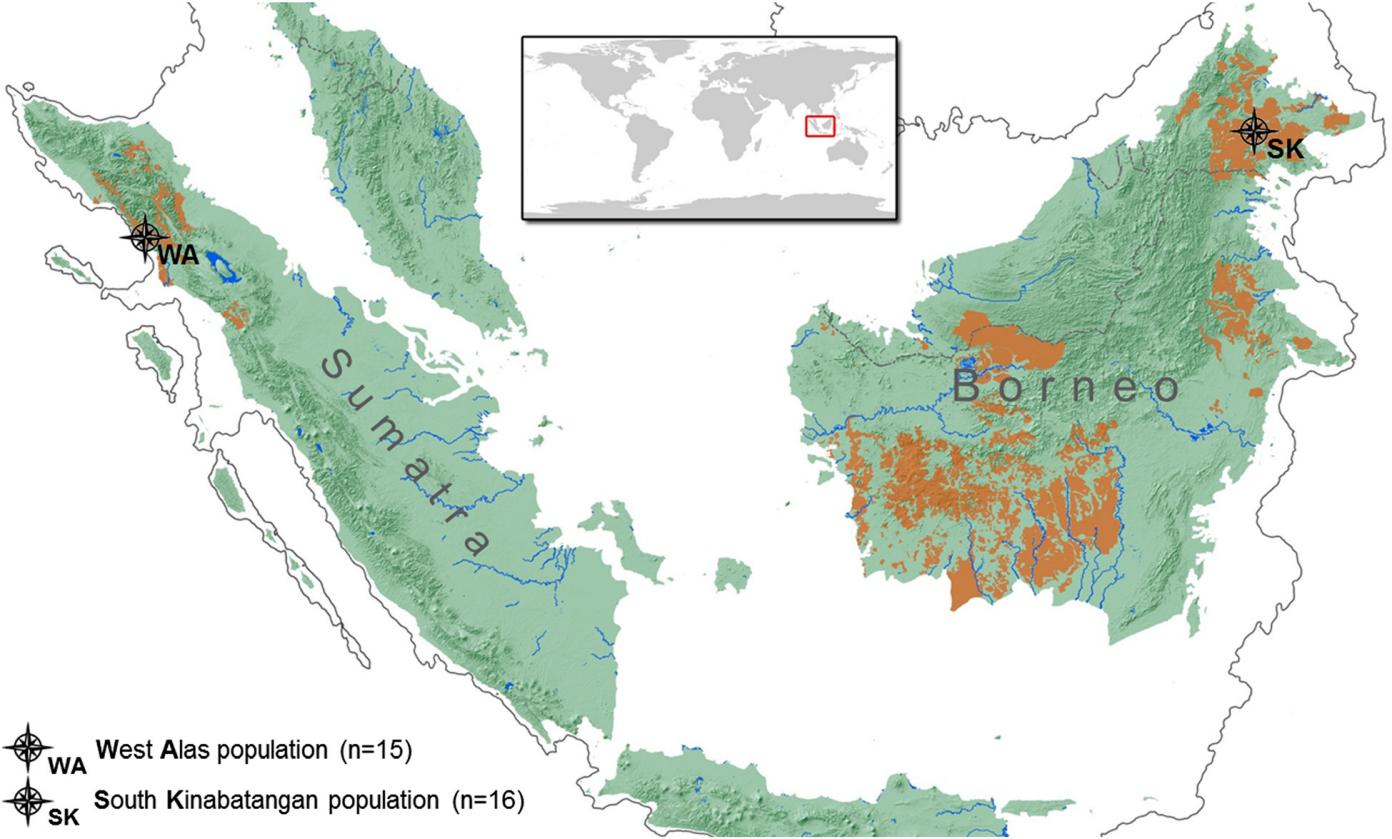
**Geographic location of the two orangutan study populations.** The areas colored in brown indicate the current distribution of orangutans.

**Figure 2:**
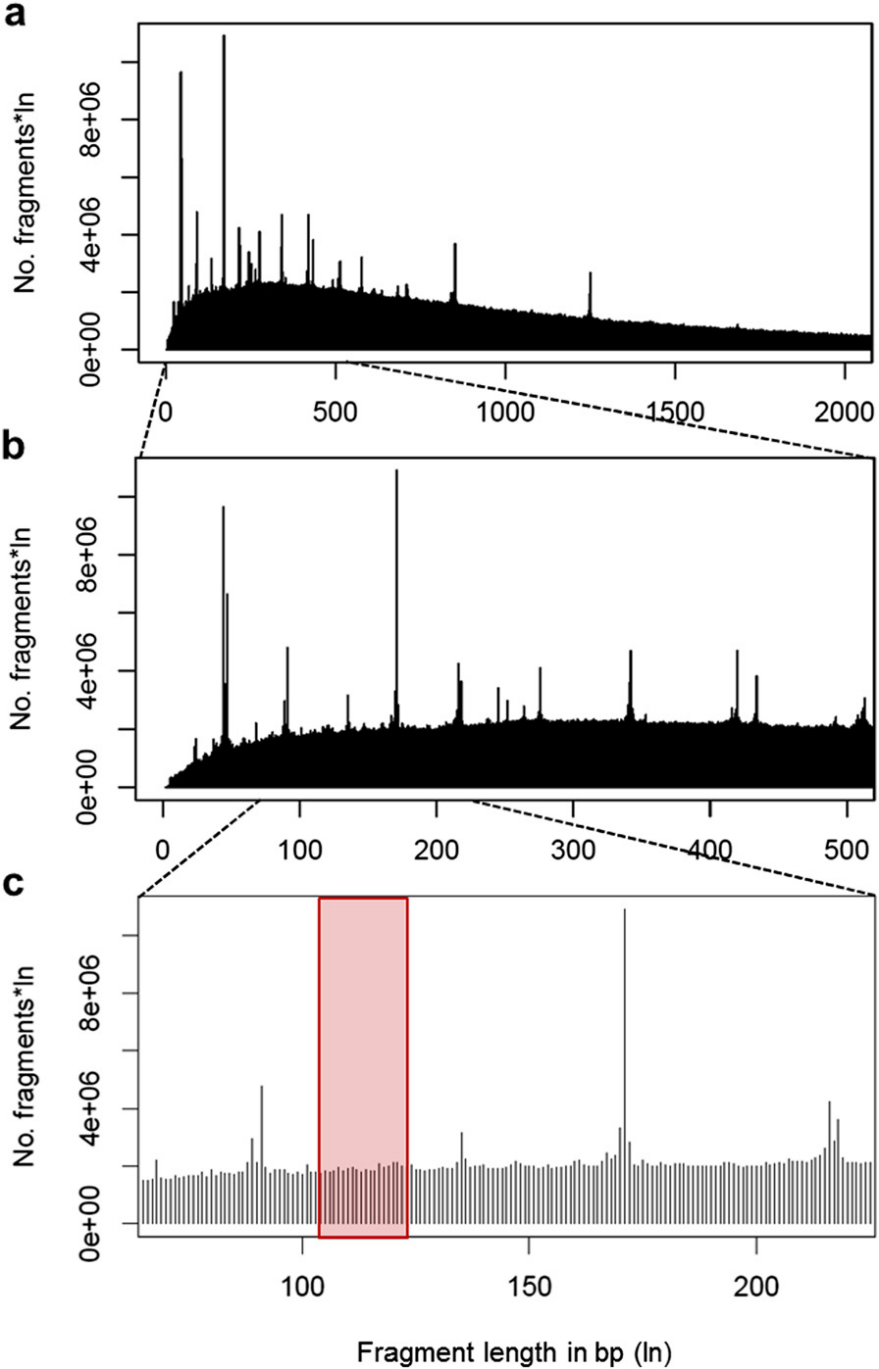
***In-silico Hae*III digest of the orangutan reference genome. **Panel **a**, **b** and **c** represent increasing levels of details. The x- and y axis show the generated fragment lengths in base pairs and the number of fragments multiplied by fragment length, respectively. Peaks are due to repetitive sequences. The isolated fragment size range (104–123 bp) is indicated in red.

We established iRRLs for 31 unrelated orangutans from two populations, the West Alas population on northwestern Sumatra (WA, *Pongo abelii*, n = 15) and the South Kinabatangan population on northeastern Borneo (SK, *Pongo pygmaeus*, n = 16; Figure 1, Additional file 2: Table S1). Based on the number of study individuals, the orangutan genome size of 3.09 Gigabases (Gb) [48], the budgeted SOLiD4 sequencing costs, and an intended 30-fold (30×) sequencing depth, we calculated our targeted degree of genome complexity reduction to be 100-fold, i.e. 1% of the genome. We carried out *in-silico* digests of the orangutan reference genome [48] with several candidate blunt-end cutters in order to identify the restriction enzyme suitable to our project needs (see Methods). In the selected size range of 104–123 bp, a *Hae*III digest yielded 305,574 predicted fragments with low repetitive sequence content (representing the desired 1.07% of the genome, Figure 2). Our *in-silico* digest demonstrated the importance of uniform fragment selection. For instance, extending the selected size range by as few as 4 bp (e.g. 100–123 bp) in all individuals would have already resulted in a 25% increase in the selected genome proportion, i.e. 1.32% of the genome with lower average coverage per site. Furthermore, a range shift of a few base pairs in some individuals in either direction would lead to a dramatic decrease in homology among the generated fragments.

In total, we obtained 675 million beads for the West Alas population and 762 million beads for the South Kinabatangan population by individually barcoding iRRLs and sequencing them on the SOLiD4 platform (Life Technologies) with paired-end chemistry. Raw sequence data were submitted to the NCBI Sequence Read Archive [BioProject: PRJNA230877; BioSamples: SAMN02439270-SAMN02439300]. Median numbers of mapped reads for each individual were 32,345,177 for the West Alas population and 43,451,986 for the South Kinabatangan population (Table 1 and Additional file 3: Table S2). The greater sequencing output for South Kinabatangan individuals is related to different performances of our SOLiD4 runs that were beyond our control. We also observed a poor performance of the F5 sequence read direction. We only considered high quality base pairs (bp_hiqual_) in downstream analyses, i.e. sites with mapping and base quality phred scores of ≥ 30, and a minimal sequence depth of 10×. Applying these stringent filters, we retained 10,930,563 bp_hiqual_ with 41× median sequence coverage for West Alas individuals and 18,186,855 bp_hiqual_ with 42× median coverage for South Kinabatangan individuals (Additional file 3: Table S2).

**Table 1:** Overview of the sequencing of improved reduced-representation libraries (iRRLs) for the West Alas (WA) and South Kinabatangan (SK) orangutan study populations

	**Pop_WA**	**Pop_SK**
	**(Sumatra)**	**(Borneo)**
No. of individuals	15	16
iRRL stacks per individual (predicted)^a^	305,574	305,574
Median iRRL target efficiency^b^	97%	86%
Total no. of beads per population	675,295,801	762,234,081
Total no. of mapped reads per population	528,081,935	646,922,204
Median no. of mapped reads per individual	32,345,177	43,451,986
% reads mapped F3/F5 (mappability)^c^	74.9/7.3	67.0/17.0
Mean no. of bp_hiqual_ per individual^d^	10,930,563	18,186,855
Median sequence coverage per individual^e^	41×	42×

^a^Predicted by *in-silico* digest of the orangutan reference genome *ponAbe2* (Sumatran) with *Hae*III. ^b^Percentage of sequenced sites that were predicted by the *in-silico* digest. ^c^F3/F5 are the sequence read directions of the paired end sequencing mode. ^d^Number of sequenced base pairs passing all high quality filters (sites used for SNP detection). ^e^GATK estimates based on bp_hiqual_.

To assess the performance of our iRRL protocol, we estimated the iRRL target efficiency as the percentage of obtained bp_hiqual_ sites which were predicted by the *in-silico* digest (= target sites). iRRL efficiency varied among individuals but was very high with a median of 97% for West Alas individuals and 86% for the South Kinabatangan individuals (Table 1 and Additional file 3: Table S2). Thus, the vast majority of sequenced high quality bases were target sites, i.e. predicted by the *in-silico* digest of the orangutan reference genome.

### Comparison of SNP discovery and genotype calling

We identified SNPs *de-novo* and called individual genotypes using three different algorithms: GATK, SAMtools, and CLC. Calls were based on the stringent bp_hiqual_ filter thresholds. For the GATK and SAMtools dataset, we also applied a minimal threshold on the genotype quality score (GQ ≥ 10). In addition, we performed identical population-based filtering for all three algorithms. We only accepted SNPs with a maximum of two alleles and genotypes meeting all quality filter criteria in at least eight individuals per population (n ≥ 16 chromosomes), allowing accurate allele frequency estimations. Applying all filters we retrieved 57,396 SNPs in the GATK dataset, 75,364 SNPs in the CLC dataset, and 24,103 SNPs in the SAMtools dataset (Table 2).

**Table 2:** Overview of SNP discovery and genotype calling using three different callers

	**GATK_v.2.5-0**			**CLC_v.5.0.1**			**SAMtools_v.0.1.19**		
	**Pop_SK**	**Pop_WA**	**Overall**	**Pop_SK**	**Pop_WA**	**Overall**	**Pop_SK**	**Pop_WA**	**Overall**
No. of SNPs	34257	40248	57396	34788	55585	75364	14494	14903	24103
No. of private SNPs	17148	23139	40287	19779	40576	60355	9200	9609	18809
% singletons	7.68	10.83	12.18	11.53	27.47	25.59	14.63	21.66	22.19
Median site heterozygosity^a^	0.267	0.250	/	0.236	0.200	/	0.266	0.231	/
Median coverage per individual	93×	70×	82×	66×	29×	48×	66×	19×	27×
	**GATK-CLC**_**intersect**_			**SAMtools- GATK**_**intersect**_			**SAMtools-CLC**_**intersect**_		
	**Pop_SK**	**Pop_WA**	**Overall**	**Pop_SK**	**Pop_WA**	**Overall**	**Pop_SK**	**Pop_WA**	**Overall**
No. of SNPs	21475	24936	37085	11325	12350	18933	9861	11310	17163
No. of private SNPs	12149	15610	27759	6583	7608	14191	5853	7302	13155
% singletons	9.91	17.98	12.82	9.99	20.53	19.37	10.54	23.08	21.60
Median site heterozygosity^a^	0.250	0.222	/	0.286	0.231	/	0.266	0.222	/
Median coverage per individual^b^	107× (65)	81× (27)	96× (37)	55× (98)	18× (98)	20× (99)	69× (76)	19× (35)	26× (46)

We required all SNPs to have a genotype call passing all stringent quality filters in a minimum of eight individuals per population (population-based filtering). The intersect datasets contain exclusively concordant genotype calls between the designated SNP callers. Pop_SK: South Kinabatangan population, Pop_WA: West Alas population.

^a^Based on the sites being polymorphic within the population.

^b^Coverage values of intersect datasets are taken from the first named SNP caller. The coverage values of the second named caller are given in brackets.

Compared to similar studies (e.g. [30,33,42,57]), median sequence coverage at SNP sites across all individuals in our datasets was extremely high (82× for GATK, 48× for CLC, and 27× for SAMtools), although coverage counts differed drastically among datasets. This discrepancy in coverage counts could be attributed to a different treatment of quality scores in read counting and/or different default parameters among the callers, since we applied identical quality thresholds to the data. The considerably lower read counts in the SAMtools dataset and potentially different prior probabilities in the Bayesian framework may be causal for the strikingly lower number of total SNPs in our SAMtools dataset.

**Figure 3:**
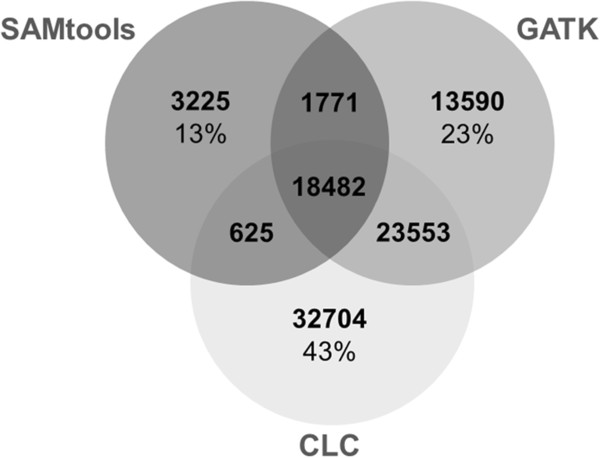
**Overlap of SNPs among the datasets obtained from three different callers.** Percentages specify the proportion of SNPs exclusively present in the particular dataset for each caller.

We observed a low overlap of SNPs among the three datasets, i.e. SNP sites present in at least two datasets irrespective of the genotype calls at the individual level (Figure 3). In total, 18,482 SNPs overlapped among all three datasets. At only 13%, the SAMtools dataset exhibited the lowest percentage of private SNPs compared to the other two algorithms (Figure 3).

Many of the non-overlapping sites were present in initial SNP discoveries, but were removed because less than eight individuals per population had a genotype call meeting all high-quality filter criteria (population-based filter). For the CLC and SAMtools dataset, genotype calls often failed the minimum coverage requirement of 10 reads. For the GATK dataset, many genotype calls did not have a sufficiently high genotype quality score.

**Figure 4:**
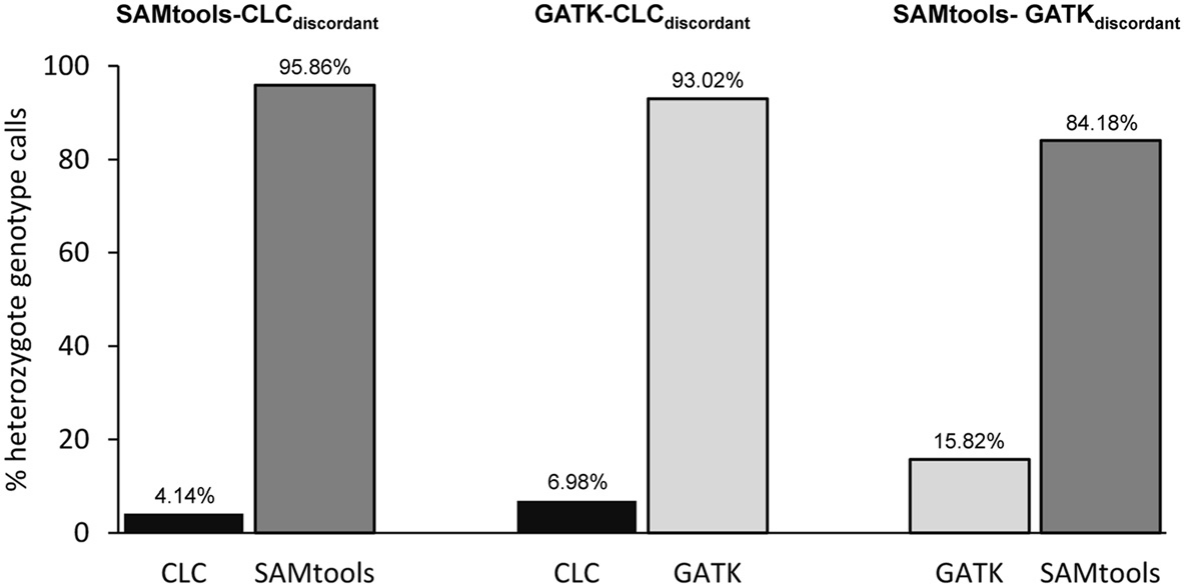
**Quantitative investigation of discordant genotype calls between pairs of SNP callers.** For the vast majority (>99.77%) of discordant genotype calls, one caller assigned a heterozygous genotype but the other caller a homozygous genotype for either of the alleles. The y-axis represents the percentage of heterozygous genotype calls in such cases. The values are median numbers across all study individuals.

For all overlapping SNPs, we evaluated the concordance of genotype assignments by comparing for each individual whether two callers produced identical genotypes. The percentage of identical genotype calls varied among individuals with median values of 97.51% for GATK-CLC, 98.32% for SAMtools-GATK, and 97.24% for SAMtools-CLC (Table 3 and Additional file 4: Table S3, Additional file 5: Table S4 and Additional file 6: Table S5). A quantitative investigation of discordantly called genotypes between the callers revealed that the vast majority (>99.77%) of these genotypes were called heterozygous by one caller but homozygous for either of the alleles by the other caller. The relative distribution of these heterozygous/homozygous genotype calls appeared to be strongly biased (Figure 4). For example, examining discordant genotype calls between GATK and CLC showed that in most cases (93.02%), GATK assigned a heterozygous genotype while CLC assigned a homozygous one. Pairwise SNP caller comparisons revealed that SAMtools had the highest tendency to call heterozygotes in such cases, followed by GATK and CLC (Figure 4).

**Table 3:** Median genotype concordance between designated SNP callers for overlapping SNP sites assessed at the individual level

	**GATK-CLC**	**SAMtools- GATK**	**SAMtools-CLC**
% same genotype called Pop_WA	96.92	98.46	96.15
% same genotype called Pop_SK	98.27	98.04	97.45
% same genotype called overall	97.51	98.32	97.24
% same genotype called overall (range)	93.59-98.38	97.08-99.26	92.46-97.82

We also created three intersect datasets by accepting only identically assigned genotypes between pairs of SNP callers (at the individual level prior to the population-based filtering). This procedure has been suggested to reduce caller-specific errors and increase specificity [18,58]. We retained 37,085 SNPs for the GATK-CLC_intersect_ dataset, 18,933 SNPs for the SAMtools-GATK_intersect_ dataset, and 17,163 SNPs for the SAMtools-CLC_intersect_ dataset (Table 2).

### Impact on biological inferences

Over all six datasets, there were more sites segregating in the Sumatran West Alas population compared to the Bornean South Kinabatangan population (Table 2). The vast majority of SNPs (70-80% depending on the dataset) were private. In addition, we observed a large percentage of singletons (Table 2). The highest number of singletons was obtained in the CLC dataset (26%) followed by SAMtools (22%) and GATK (12%). Median site heterozygosity was always higher for the South Kinabatangan population than for the West Alas population.

**Figure 5:**
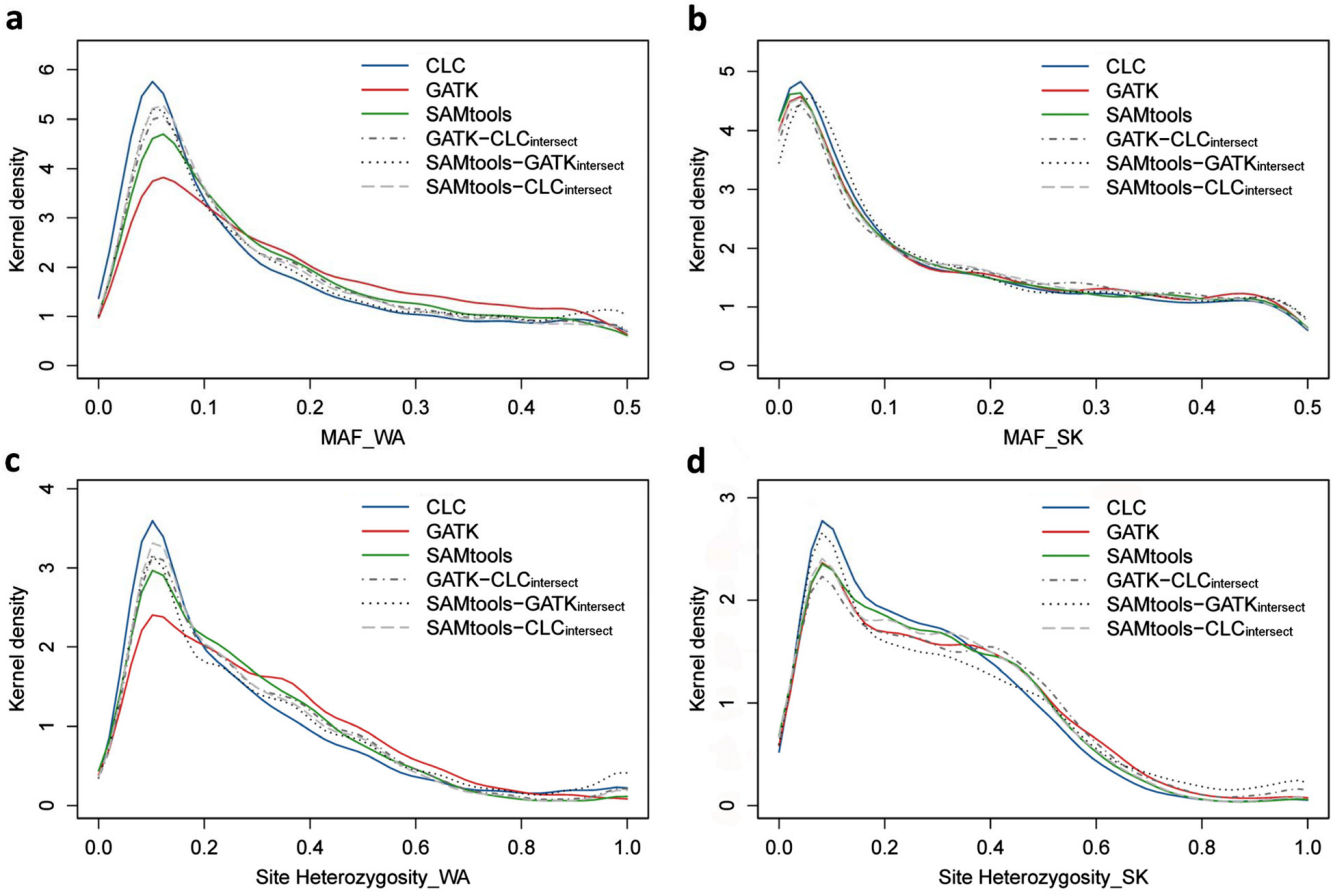
**Kernel density distributions of minor-allele frequency and site heterozygosity using the different SNP datasets.** For each of the six SNP data sets (CLC, GATK, SAMtools, GATK-CLC_intersect_, SAMtools-GATK_intersect_, and SAMtools-CLC_intersect_) we computed the minor-allele frequency (MAF) for the Sumatran (WA) and Bornean (SK) individuals (panels **a** and **b**, respectively), and site heterozygosity for WA and SK (panels **c** and **d**, respectively).

To investigate the potential impact of the different SNP datasets on biological downstream analyses, we calculated three important statistics. (i) Kernel-density distributions for site heterozygosity and (ii) minor allele frequency were not identical among the SNP datasets (Permutation test of equality, *p* < <0.001, Figure 5). From a qualitative point of view, differences in kernel density distributions among all six datasets were especially pronounced for the West Alas population (Figure 5a,c) for which median sequence coverage was lower compared to the South Kinabatangan population. Nevertheless, it is striking that we obtained these differences despite a stringent minimal read cut-off of 10 reads and 29× (CLC value) medium sequence coverage. For example, the CLC dataset consisted of the largest proportion of low frequency alleles. In contrast, GATK called more variants at mid-frequency and showed higher overall heterozygosity levels.

**Figure 6:**
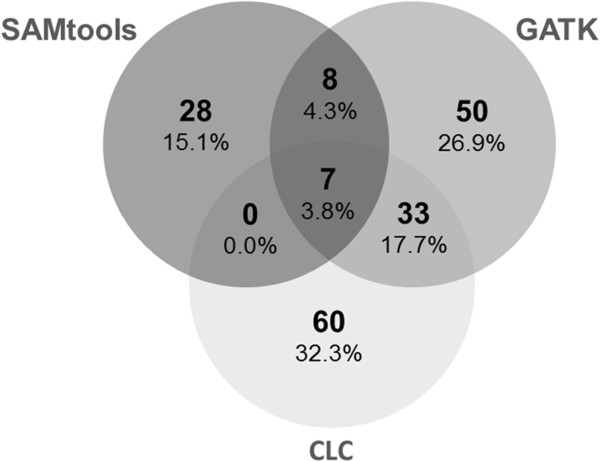
**Overlap of outlier regions among SNP datasets in genome-wide scans for positive selection.** For all SNP datasets we performed sliding-window analyses (100 kb window, 25 kb step size) of the absolute allele-frequency differential (*D*) between the SK and WA population. All windows with an average window *D* > 0.95 were considered as outliers, i.e. candidate regions for selective sweeps. Percentage values are given in relation to the total number of outlier windows.

To evaluate the impact of the SNP dataset differences on genome-wide scans for signatures of natural selection, we performed (iii) sliding-window analyses (100 kb windows, 25 kb step size) to identify signals of putative selective sweeps based on population differentiation. We used the allele-frequency differential (*D)* to measure population differentiation. We arbitrarily defined outlier regions as windows with an average population differentiation *D* > 0.95 (covered by at least 2 SNPs). The overlap of outlier windows among datasets was low. Only 3.8% of all detected outlier windows were identical among all three single-caller datasets (Figure 6), which improved to 13.5% when intersect datasets were used (Additional file 1: Figure S3).

### Genotype validations

To determine genotype accuracy, we validated 63 genotypes from a subset of 58 SNPs overlapping among datasets by classical Sanger sequencing. We picked SNPs with the only requirements that a minimum of ten individuals per population had an assigned genotype and that at least one individual showed a conflicting genotype call between GATK/SAMtools and CLC. Because all validated genotypes were identical between GATK and SAMtools, we did not distinguish between the two for this analysis, but rather focused on the difference between probabilistic (GATK and SAMtools) and hard-filtering (CLC) callers.

Our results show that GATK/SAMtools clearly outperformed CLC, with a correct genotype assignment in 83% of the conflicting calls (Table 4). GATK/SAMtools calling accuracy was especially high for singletons (92% true in GATK/SAMtools, 8% true in CLC) and for genotypes that were according to GATK/SAMtools homozygous for either of the two alleles but heterozygous according to CLC (89% true in GATK/SAMtools, 11% true in CLC). We also verified the genotype accuracy of identical calls and found 4 miscalled genotypes out of 114 (3.5%).

**Table 4:** Overview of genotype validations at overlapping SNP sites

	**SNPs validated**	**Genotypes validated**	**True CLC**		**True GATK/SAMtools**	
**Category**			**n**	**%**	**n**	**%**
***Discordant calls***^***a***^						
Singleton site determined by GATK/SAMtools^b^	8	8	1	12.5	7	87.50
Singleton site determined by CLC^b^	4	4	0	0.00	4	100
Homozygote with GATK/SAMtools but heterozygote with CLC	23	28	3	10.71	25	89.29
Heterozygote with GATK/SAMtools but homozygote with CLC	23	23	7	30.43	16	69.57
**Total**	**58**	**63**	**11**	**17.46**	**52**	**82.54**
***Concordant calls***^**c**^						
**Total**	**53**	**114**	**110 (96.49%)**			

^a^Overlapping SNP sites but discordant genotype assignments. ^b^Loci were exclusively counted in this category without considering them in the homo- or heterozygote categories below. ^c^100 of the 114 genotypes were validated from the same sites used to validate the discordant genotypes. The remaining 14 genotypes were validated from 14 SNPs chosen randomly from the GATK-CLC_intersect_ dataset (exclusively identical genotype calls).

### Characteristics of SNP callers

GATK seemed to be conservative in calling singletons and low frequency alleles in our dataset, as it exhibited the lowest proportion of singletons among all SNP datasets. Yet, among all datasets, GATK had the highest medium site heterozygosity. It appears that GATK slightly overestimates mid-frequency alleles, because our genotype validations revealed that in 30% of the cases where GATK called a heterozygous and CLC a homozygous genotype, CLC was correct. Thus, our results suggest that with increasing minor allele frequency, GATK starts calling alternative alleles more aggressively due to the population prior in multi-sample analysis.

It appears that CLC generally underestimates heterozygosity. The CLC dataset consisted of an excess of singletons, suggesting that CLC called sequence errors as a genetic variants to a greater extent. Thus, the CLC dataset contained the lowest overall site heterozygosities among all datasets. Detailed investigation of discordantly called genotypes revealed that almost all of these genotypes were homozygous with CLC, but heterozygous with the other callers. To our surprise, CLC largely miscalled genotypes as heterozygous which were correctly assigned as homozygous by GATK (89% correct by GATK).

Our results indicate that SAMtools is more restrictive in SNP calling than GATK and CLC. The SAMtools dataset consisted of considerably fewer SNPs than GATK and CLC, but the degree of overlap with the other datasets was much higher than for the other datasets. SAMtools showed the highest tendency to assign heterozygous genotypes in cases of discordantly called genotypes among callers. For example, the few discordantly called genotypes were strongly biased in that 84% were heterozygous with SAMtools, but homozygous with GATK.

## Discussion

Our study provides a framework for the generation of genome-wide SNP datasets for population genomic studies, from laboratory procedures to bioinformatics, which is widely applicable in non-model species. We present an improved protocol for highly efficient and more precise reduced genome complexity sequencing that simultaneously allows discovery of novel SNPs and genotyping. Using data generated from 31 wild-born orangutans from two populations, we observed significant inconsistencies among three commonly used SNP callers (*CLC Genomics Workbench, GATK Unified Genotyper* and *SAMtools*). These inconsistencies among the SNP datasets led to strong disagreement in outliers detected in scans for signatures of natural selection. This shows the potential impact on downstream biological analyses and emphasizes the need to critically evaluate the accuracy of SNP and genotype calling in population genomic studies.

We present a refined iRRL method presenting an improvement of the approach by van Tassel *et al*. [19]. Several key modifications greatly enhanced the effectiveness of genotyping-by-sequencing, as measured by target sequence efficiency. Target sequence efficiency was high because we focused on laboratory procedures to obtain homologous sequences across individuals, i.e. reproducible fragment generation and precise size selection. To our knowledge, these procedures do not seem to have received sufficient attention in the literature, probably because most studies pooled individuals to develop SNP markers [26,32,33] without the direct aim of estimating allele-frequencies.

The importance of uniform fragment selection is well illustrated by our *in-silico* digests of the orangutan reference genome. An imprecise isolation of fragments would have led to a substantial change in the overall composition of fragment libraries across samples. This in turn would have caused a substantial increase in missing genotypes because of significantly reduced overlap of homologous fragments. Thus, accurate size selection and generation of uniform fragments to achieve high sequences homology are paramount in producing high-quality RRLs that maximize the amount of biological information.

The higher and more constant target sequence efficiencies for Sumatran West Alas individuals (median 97%) compared to Bornean South Kinabatangan individuals (median 86%) were most likely caused by carrying out the initial *in-silico* digest, which predicted our target sites, on the Sumatran reference genome. Since Sumatran and Bornean orangutans diverged more than 400,000 years ago, [48,53,56], Bornean orangutans will inevitably exhibit more mutations at restriction sites.

We also improved previous RRL approaches by minimizing the loss of DNA during purification steps, thus facilitating single-sample library construction. Economical handling of DNA is particularly relevant when studying species for which sample quantity is a limiting factor, which is the case for most wild animal populations. A high DNA recovery rate during purification steps is especially important when dealing with low template amounts (<100 ng), where DNA loss will be disproportionately higher for technical reasons, and/or targeting only a small fraction of the genome. So far, these problems have been circumvented by pooling samples. Our DNA recovery rate of >95% in the purification steps is considerably higher than obtained through conventional methods using extractions from gels and/or silica columns [<80%; QIAquick Spin Handbook Qiagen].

From a bioinformatical perspective, we demonstrate that different SNP callers lead to substantially different SNP datasets, in spite of applying rather conservative quality filters. For example, we applied a phred-scaled mapping and base quality threshold of ≥ Q30, corresponding to an error probability of ≤0.1%. In contrast, other studies only apply Q20 (1.0% error probability) [18,26,59,60]. Furthermore, our median sequence coverage of 41× (minimal cut-off of 10 reads) is substantially higher than that found in other studies, in which sequencing depth is usually between 6-16× with lower cut-off values than used in this study [30,32,33,42,57].

There are three main reasons for the conspicuous differences among the SNP datasets. First, the SNPs dropping out because of our population-based filtering were different among the GATK, SAMtools and CLC datasets. Second, although we used identical mapped short reads and filtering criteria on the raw data to call SNPs and genotypes, we cannot exclude a potential influence of the poor F5 sequence read performance due to specific internal filters of SNP callers. Third and most importantly, some differences will arguably be related to the conceptually very different methods of SNP identification and genotype assignment [18,36-38].

Intersect strategies have been proposed to reduce caller-specific errors [18,58]. The estimated genotype accuracy of 96.5% of intersected genotypes is higher than in comparable studies that use only one caller (e.g. 47-84% [26,42,61]; 89-95% [19,31,32,62]). Yet, most of these studies actually only verified the polymorphic state of SNPs but not individual genotype calls. Thus, the true genotype error rate in these studies is almost certainly higher than estimated.

The intersect strategy seems to be appealing because false-positive assignments should be minimized. However, it is inevitably less sensitive towards SNP discovery [18]. The appropriate strategy and filter stringencies for each study depend on the specific needs of downstream analyses. Nonetheless, apart from higher false-negatives rates, as observed in our dataset, intersecting genotype calls might also introduce non-random biases. More detailed investigations will be required to fully appreciate the consequences of intersecting strategies.

Among all datasets, the general patterns tend to agree with previous detailed studies on orangutan population genetics and demographic history. For instance, the higher number of singletons and low-frequency alleles we observe in the Sumatran West Alas population is in agreement with previous studies using conventional genetic markers (mitochondrial DNA, microsatellites) [52-54]. Furthermore, the slightly higher site heterozygosities in the South Kinabatangan population are also in agreement with previous studies using conventional genetic markers [52,63-65].

Many downstream analyses in population genomics, such as selection tests or demographic inferences rely on the allele-frequency spectra [41]. Thus, biological conclusions drawn from such analyses may well change depending on which SNP caller has been used. This possibility is illustrated by the extremely low overlap of identified outlier regions in our sliding-window analyses to detect selective sweeps based on population differentiation.

Apart from reliable SNP analysis, the accurate characterization of the allele-frequency spectra is mainly influenced by three sources of bias. First, allele frequencies will not be representative of the population if there is a sampling bias [9,66,67]. To address this issue and reduce this bias, we carefully selected study animals and verified population origins. By contrast, genomic studies often rely on zoo animals with unknown population provenance (if wild-born) or apply a limited sampling schema (e.g. [48]), and thus there are likely inherent sampling biases.

Second, the discovery of SNPs in a subset of individuals for subsequent genotype calling in an extended sample set will lead to ascertainment bias [1,17,27]. The degree of ascertainment bias depends on the representativeness of the sampling scheme of individuals used for the initial SNP discovery [67]. Especially in population and conservation genomics, ascertainment bias is a serious problem when assessing, for instance, genetic diversity. Low-frequency variants will be underestimated and a systematic bias will be introduced [17]. The key strength of reduced genome complexity approaches is that this form of ascertainment bias can be minimized by the genotyping-by-sequencing principle.

Third, it is biologically relevant to also capture rare alleles, which is the reason why we established individual libraries (i.e. no pooling of samples). Low-frequency alleles are important in estimates of demographic parameters [68] and studies of positive [69] and purifying selection [70].

The framework provided in this study will be valuable to generate genome-wide SNP datasets in the emerging fields of population, conservation and landscape genomics. Our iRRL protocol is part of a growing suite of sequencing methods, which have completely changed study designs and hold great promise for studies of ecology and evolution in diverse species. The strength of reduced-genome-complexity RRL methods is that they can be applied to any DNA-based life form, opening up the field of population genomics to smaller research groups studying organisms for which large-scale genetic data is not yet available. Until high-throughput sequencing becomes more affordable and bioinformatical advances allow routine whole-genome re-sequencing of populations, we expect that reduced-genome-complexity approaches will remain essential for population genomic studies particularly in non-model organisms with large genomes.

## Conclusions

We generated SNP datasets for 31 wild-born orangutans from two populations representing the first effort of large-scale SNP discovery and genotyping of orangutans with known population provenance. In the field of population genomics, researchers need to exert caution when generating genome-wide SNP datasets. We show that accurate generation of homologues fragments in reduced-genome-complexity sequencing is paramount, especially for pooled samples with no control for missing genotypes in the estimation of allele frequencies. We present an improved RRL protocol (iRRLs), which allows sampling only a fraction of the genome with maximized sequence overlap among individuals. The scale and efficiency achieved with our iRRL protocol demonstrates its suitability to generate genome-wide SNP datasets. Our direct comparison of three popular SNP callers demonstrated that depending on the calling algorithm, sequence depths and filtering criteria, substantially different SNP datasets are obtained that will affect downstream analyses and thus might have a substantial effect on biological conclusions. When only applying a single SNP caller, we advise to use a probabilistic algorithm and call genotypes in a multi-sample mode. In our study, the Bayesian framework of the *Unified Genotyper* of the *GATK* showed a higher sensitivity in discovering SNPs than the framework of *SAMtools* with similar genotype calling accuracy.

## Methods

### DNA samples

We sampled two orangutan populations, one from Borneo and one from Sumatra (Figure 1). To obtain sufficient amounts of high-quality DNA, we collected blood samples from rehabilitant wild-born orangutans. We sampled 15 individuals from the West Alas population (WA, *Pongo abelii*, northwestern Sumatra) at the Batu Mbelin Quarantine Center of the Sumatran Orangutan Conservation Programme, and 16 individuals from the South Kinabatangan population (SK, *Pongo pygmaeus morio*, northeastern Borneo) at the Sepilok Orangutan Rehabilitation Centre, Shangri-La's Rasa Ria Resort Sanctuary and Lok Kawi Wildlife Park in Sabah. Whole blood samples were taken during routine veterinary examinations and stored in EDTA blood collection tubes at −20°C. The collection and transport of samples were conducted in strict accordance with Malaysian, Indonesian and international regulations. Samples were exported from Malaysia and Indonesia to Switzerland under the Convention on International Trade of Endangered Species in Fauna and Flora (CITES) permit numbers 4872/2010 (Sabah, Malaysia) and 06968/IV/SATS-LN/2005 (Indonesia), respectively. Detailed information on the sampled individuals is provided in Additional file 2: Table S1. We verified the individual’s population origin by genetic assignment tests and Bayesian clustering algorithms as described in the Additional file 1.

To minimize DNA shearing, we avoided repeated thawing and freezing of samples and used only wide-bore tips and avoided vortexing during DNA extraction. Genomic DNA was extracted using the Gentra Puregene Kit (Qiagen) according to the manufacturer’s instructions, including RNase treatment, but with the following modifications for clotted blood: we added twice the amount of Cell Lysis Solution as well as 7 μl of Proteinase K (20 mg/ml, Promega) per 100 mg blood clot to the samples, followed by incubation for 3 hours at 55°C in a slowly revolving overhead rotator. If the solution still appeared to be viscous after this treatment, we increased incubation time and added more Proteinase K as required until complete liquefaction. We also used twice the recommended amount of Protein Precipitation Solution and incubated samples on ice for 10 minutes after addition of the solution to promote protein precipitation. DNA pellets were eluted in ddH_2_O instead of DNA Hydration Solution (Qiagen) to facilitate DNA concentration using a SpeedVac vacuum centrifuge (Savant).

### Reduced-representation libraries construction

We performed *in-silico* digests of the orangutan reference genome (*ponAbe2*[48]) to evaluate a suitable restriction enzyme to construct iRRLs using custom perl scripts. We tested 23 commercially available Type II DNA blunt-end cutters (4–6 bp recognition sites) in multiple combinations (Additional file 7: Table S6). Selection criteria were: (i) target size range 70–200 bp, (ii) number of fragments predicted in size range corresponding to ~1% of the genome, and (iii) low repetitive element content. We chose *Hae*III because in the size range of 104–123 bp, *Hae*III did not produce obvious repetitive elements based on visual inspection of the fragment distribution profile (Figure 2), and covered ~1% of the genome. The enzyme *Hae*III has also been selected in previous studies [19,31,60], and thus might be a good candidate enzyme for reduced-genome-complexity sequencing in general.

In cases where there is no reference genome available, the evaluation for a suitable enzyme could also be carried out in the laboratory, for example by analyzing the fragment distribution of digested genomic DNA using high resolution electrophoresis (e.g. Agilent 2100 Bioanalyzer). These instruments offer tools to estimate the represented genome proportion of fragments within a given size range.

We established iRRLs for each individual by digesting 20 μg of genomic DNA with 200 units of *Hae*III (50,000 U/ml, New England Biolabs) in a total volume of 32 μl. Digests were run on high-resolution Spreadex EL400 Wide Mini S-2 × 13 gels with M3 size marker in a SEA 2000 electrophoresis chamber (all Elchrom Scientific, Switzerland) in 1× TAE buffer at 120 Volt for 147 min, keeping temperature constant at 55°C to ensure reproducibility of fragment migration. The running time was the evaluated optimum for the target size range using the ELQuant Software (http://www.elchrom.com). Each digest was equally distributed in two separate wells to avoid DNA overloading. We stained gels with GelRed (Biotium) and excised fragments between 104 bp and 123 bp on a UV-transilluminator using a long-bladed sharp knife, keeping UV exposure as short as possible.

DNA fragments were recovered by electro elution to achieve high DNA recovery rate (>95%). For this, we prepared dialysis membranes (Carl Roth, 1785.1 Dialysierschlauch Visking) of approximately 5 cm width, which we sealed on one side with a plastic clip (Carl Roth, H277.1 Verschlussklammer). We filled each dialysis membrane with 1 ml of 1× TAE buffer and placed gel slices in the membrane in the same running orientation as in the electrophoresis run (illustrated in Additional file 1: Figure S3). We closed the dialysis membrane with a second plastic clip and avoided trapping any air bubbles inside the membrane. Packages were then placed in an SEA 2000 electrophoresis chamber filled with 1× TAE buffer. We applied 90 Volts for 100 minutes, followed by 1 minute of reverse polarity to detach DNA from the wall of the membrane. We gently massaged the packages to mix the eluted DNA in the buffer. After this, we carefully opened one of the clips to gently pipet out the buffer containing the eluted DNA. The DNA was purified using the MinElute PCR Purification Kit (Qiagen). This way, we obtained between 2 and 20 ng of DNA per sample. Individual barcoding of iRRLs and SOLiD sequencing library preparation was performed according to the SOLiD ChiP-Seq protocol step 11 (Applied Biosystems, 2010), which had been optimized for low template quantities (e.g. Agencourt AMPure XP beads for purification steps). We restricted library amplification to six PCR cycles only, so as to minimize the risk of over-amplification. After library quality control on an Agilent Bioanalyzer 2000, we normalized samples and sent pooled libraries to the Functional Genomics Center Zurich, Switzerland (FGCZ) for sequencing on a SOLiD 4™ System with paired-end (50/35) chemistry (Life Technologies).

### SNP discovery and genotype calling

Raw sequence reads were processed and mapped to the orangutan reference genome *ponAbe2*[48] using the SOLiD LifeScope v.2.5.1 package (Life Technologies) according to their guidelines. We used Picard v.1.57 [http://picard.sourceforge.net/] to merge mapping files for each individual from different SOLiD runs and adjust read group headers. We called SNPs using three different programs as described below.

We performed simultaneous multi-sample SNP and genotype calling with the Unified Genotyper of the GATK v.2.5-0 [37,38] with the following thresholds: phred-scaled mapping and base qualities ≥ 30 (‘-mmq 30 -mbq 30’). We filtered out low-quality genotypes (GQ < 10) and genotypes covered by less than 10 or more than 1000 reads (‘-minGQ 10 -minDP 10 -maxDP 1000’) using VCFtools v.0.1.9 [70]. Sites which were homozygous after this filtering were removed. Finally, we disregarded sites with more than two alleles and only retained sites with a genotype call for a minimum of eight individuals per population that had passed all quality filters applying custom R scripts.

As a second probabilistic caller, we used SAMtools v.0.1.19 [36] to call SNPs and genotypes in all individuals simultaneously. We applied the same filter thresholds as for the GATK dataset and used defaults settings otherwise (except for deactivating the base alignment quality realignment with the -B parameter: ‘samtools mpileup -q 30 -Q 30 -B’). Post-filtering of SNP and genotype calls was conducted as for the GATK dataset.

As an alternative non-probabilistic approach, we discovered SNPs with the quality-based variant detection tool of the CLC Genomics Workbench v.5.0.1 (CLC bio) following the same quality requirements as applied in the GATK/SAMtools calls. Since the CLC version we used did not offer multi-sample calling (i.e. analyzing all individuals simultaneously) at the time of this study, we detected SNPs for each individual separately and merged the SNP data subsequently using R scripts. In this merged dataset, a missing call for an individual for a certain SNP position could arise either because this individual is homozygous for the reference allele or because this site was not sequenced. To obtain this information for all missing genotypes, we used SAMtools v.0.1.12a [36]. We called genotypes according to common practice, applying fixed cut-off rules based on read counts [18] with *ad-hoc* R scripts. Sites with an alternative allele frequency between 0-15% were called homozygous for the reference allele, sites with an alternative allele frequency between 20-80% as heterozygous, and sites between 85-100% alternative allele frequency as homozygous for the alternative allele. To be conservative, we denoted sites with borderline alternative allele frequencies (i.e. 15-20% and 80-85%) as ‘N’. We only accepted sites with a maximum of two alleles and covered by minimal eight individuals per study population, as we had done for the GATK and SAMtools datasets.

Finally, we used custom R scripts to intersect the GATK, SAMtools and CLC genotype calls for each individual at all sites (without the population-based filters) only retaining identical genotype calls. After merging the individual data, we again excluded sites with more than two alleles and genotypes in less than eight individuals per population as performed with the other datasets (population-based filtering). Note that further filters could be applied for SNP and genotype calling from high-throughput sequencing data such as filtering clusters of SNPs (for a list see Supporting Information of Auton *et. al.*[47])

### SNP and genotype validation

To assess genotype accuracy between the probabilistic callers and CLC and estimate the error rate of identical genotype calls, we validated 180 genotypes by classical Sanger sequencing. We randomly picked 58 overlapping SNPs with the only requirements that a minimum of 10 individuals per population had a genotype called, and that at least one individual showed a conflicting genotype call. We validated genotypes of several individuals at those SNPs, which appeared to belong to three different classes: (i) homozygote genotype with GATK/SAMtools but heterozygote with CLC, (ii) heterozygote genotype with GATK/SAMtools but homozygote with CLC, (iii) identical genotype call. We also validated (iv) singleton sites (only one alternative allele called in the entire dataset) that were determined by only one of the callers through Sanger-sequencing of the individual that exhibited the singleton. Additionally, to specifically investigate the genotype accuracy of SNPs present in the intersect data of all three callers, we randomly picked an additional 14 SNPs from this dataset. BEDtools v.2.16.2 [71] was used to extract the DNA sequences 400 bp downstream and upstream of the targeted SNPs from the orangutan reference genome *ponAbe2*. PCR primers flanking the SNPs were designed with Primer3 [72] (Additional file 8: Table S7). We verified genotypes by sequencing PCR products on a 3730 DNA Analyzer (Applied Biosystems). Details on PCR conditions, cycle sequencing and data analyses are provided in the Additional file 1.

### Statistical analyses

We considered all sites with mapping and base quality phred scores of ≥30, and a minimal sequence depth of 10 as high-quality base pairs (bp_hiqual_). We estimated the target efficiency of our iRRL protocol by calculating which percentage of the actually sequenced bp_hiqual_ was predicted by our *in-silico* digest of *ponAbe2* with *Hae*III (=target sites). Furthermore, for each SNP and population we calculated the observed site heterozygosity as the number of individuals carrying both alleles divided by the total number of called genotypes in this population. Kernel density plots of the minor allele frequency and site heterozygosity distributions were drawn in R with the ‘sm’ package [73]. We assessed the significance of equality of the density estimates among the different datasets with the ‘sm.density.compare’ function with 10,000 permutations.

In addition, we performed sliding-window analyses for each dataset to detect selective sweeps in the genome based on population differentiation using custom R-scripts. For all SNPs we estimated population differentiation using allele-frequency differentials, defined as:  *D* = ∑ [*abs*(pSK − pWA) + abs(qSK − qWA)]/2, where p and q denote the frequencies of the two alleles for each SNP. We scanned each chromosome ('chrXY_random' excluded) and calculated for each window (100 kb window size, 25 kb step size) the average *D* of all SNPs. We arbitrarily defined outlier regions as windows with an average population differentiation *D* > 0.95 (covered by at least 2 SNPs).

### Availability of supporting data

The raw sequence data to this article is available in the NCBI Sequence Read Archive, BioProject: PRJNA230877; BioSamples: SAMN02439270-SAMN02439300.

## Abbreviations

SNP: Single-nucleotide polymorphism; RRL: Reduced representation library; iRRL: Improved reduced-representation library; WA: West Alas population (Sumatra); SK: South Kinabatangan population (Borneo); bphiqual: Sequenced bases with mapping and base quality phred scores of ≥30 and a minimal sequence depth of 10; GATK: *Unified Genotyper* of the *Genome Analysis Toolkit* (Broad Institute); CLC: SNP caller of the *CLC Genomics Workbench* (CLC bio); bp: Base pair; Gb: Gigabases; D: Allele frequency differential; MAF: Minor allele frequency.

## Competing interests

The author(s) declare that they have no competing interests.

## Authors’ contributions

MPG, MK conceived and coordinated the study with input from CPvS. MPG designed and performed experiments. KNS, AN, and RHSK contributed to the experimental design. BG, MPG, MK, RS, IS, LNA, LC, and CPvS provided genetic samples. AN and NA supported the population genetic assessment of study individuals. BN contributed ideas and reagents. RB and MPG conducted *in-silico* analyzes. AP contributed reagents and performed sequencing. RB carried out short read mapping. MPG performed bioinformatical analyses. KNS supported the bioinformatical analyses. MPG, MK, and NA wrote the manuscript. KNS, AN, BN, RHSK, LC, RB, RS, BG, and CPvS critically revised the manuscript. All authors read and approved the final manuscript.

## Supplementary Material

Additional file 1 **Describes the population genetic assessment of study individuals and the genotype validations through Sanger-sequencing in detail.** Contains **Figure S1.** which shows the STRUCTURE analysis results, **Figure S2.** which shows an example image of the high precision excision of target fragments from Spreadex gels, and **Figure S3.** which illustrates the low overlap of outlier regions among SNP datasets in genomic screens for positive selection. Click here for file

Additional file 2: Table S1 List of study individuals. Click here for file

Additional file 3: Table S2 Basic sequencing and mapping statistics for all study individuals. Click here for file

Additional file 4: Table S3 Comparison of the SNP and genotype calling of GATK and CLC for each individual. Click here for file

Additional file 5: Table S4 Comparison of the SNP and genotype calling of GATK and SAMtools for each individual. Click here for file

Additional file 6: Table S5 Comparison of the SNP and genotype calling of SAMtools and CLC for each individual. Click here for file

Additional file 7: Table S6 List of tested restriction enzymes. Click here for file

Additional file 8: Table S7 PCR primer sequences used for the genotype validations. Click here for file
