# Supplementary material for: Generation of SNP datasets for orangutan population genomics using improved reduced-representation sequencing and direct comparisons of SNP calling algorithms
Source: BMC Genomics. 2014 Jan 10;15:16. doi: 10.1186/1471-2164-15-16 (PMC3897891; doi:10.1186/1471-2164-15-16)
Supplement: Additional file 1 — Describes the population genetic assessment of study individuals and the genotype validations through Sanger-sequencing in detail. Contains Figure S1. which shows the STRUCTURE analysis results, Figure S2. which shows an example image of the high precision excision of target fragments from Spreadex gels, and Figure S3. which illustrates the low overlap of outlier regions among SNP datasets in genomic screens for positive selection. [file 1471-2164-15-16-S1.pdf]

Supporting Information

Population genetic assessment of study individuals

Our detailed knowledge of the orangutan population structure through previous studies [1-5] on both Borneo and Sumatra, allowed us to assign individuals to their population of origin. We genotyped the animals of the current study at the same 27 highly polymorphic microsatellite makers used in previous studies [6, 7]. We then used the program STRUCTURE v2.3.3 [8] to analyze the study animals for this study together with genotype data from 219 individuals from previous studies [1-5] that represent all major genetic clusters on both islands. For each individual we estimated the membership coefficient Q [8] of belonging to a particular cluster (Figure S1). Details on the analysis and the general observed population structure can be found in Nater [5]. The highest hierarchical level clearly separates Bornean and Sumatran individuals. Analyzing each island separately, three distinct clusters seem to best describe the structure observed in the variation analyzed on Sumatra and five clusters on Borneo. All individuals from this study show high membership to the South Kinabatangan and West Alas cluster, which had also been confirmed by phylogenetic analyses of mitochondrial DNA gene sequences in Nater et. al. [2] .

SNP validation

All PCR reactions were performed in a 10 µl reaction volume containing 5 ng of template DNA, 0.1 µM of each primer, 0.2 mM dNTPs, 1 x Phire PCR Buffer and 0.1 units Phire Hot Start II DNA Polymerase (both Finnzymes). PCR conditions were as follows: initial denaturation at 98°C for 30 s, followed by 35 cycles of 98°C for 5 s, 62°C for 10 s, 72°C for 15 s, and final extension at 72°C for 2 min. Direct cycle sequencing was performed with 0.5 µl PCR product in a 10 µl reaction volume containing 1x sequencing buffer (400 mM Tris, 10 mM $MgCl_2$, pH 9.0), 0.4 µM forward primer and 0.3 µl BigDye Terminator v3.1 (Life Technologies). Cycle sequencing conditions were as follows: initial denaturation at 95°C for 45 s, 30 cycles of 95°C for 30 s, 52°C for 20 s, 60°C for 2 min. Samples were sequenced on a 3730 DNA Analyzer (Life Technologies). We aligned generated sequences with the reference genome sequence *ponAbe2* [9] using the SeqMan program of the Lasergene 8 software package (DNASTAR) and visually called genotypes for the target SNP position based on the trace data.

References

1. Nater A, Arora N, Greminger MP, van Schaik CP, Singleton I, Wich SA, Fredriksson G, Perwitasari-Farajallah D, Pamungkas J, Krützen M: **Marked population structure and recent migration in the critically endangered Sumatran orangutan (Pongo abelii)**. *Journal of Heredity* 2013, **104**(1):2-13.
2. Nater A, Nietlisbach P, Arora N, van Schaik CP, van Noordwijk MA, Willems EP, Singleton I, Wich SA, Goossens B, Warren KS *et al*: **Sex-biased dispersal and volcanic activities shaped phylogeographic patterns of extant orangutans (genus: Pongo)**. *Molecular Biology and Evolution* 2011, **28**(8):2275-2288.
3. Nietlisbach P, Arora N, Nater A, Goossens B, Van Schaik CP, KrÜTzen M: **Heavily male-biased long-distance dispersal of orang-utans (genus: Pongo), as revealed by Y-chromosomal and mitochondrial genetic markers**. *Molecular Ecology* 2012, **21**(13):3173-3186.
4. Arora N, Van Noordwijk MA, Ackermann C, Willems EP, Nater A, Greminger M, Nietlisbach P, Dunkel LP, Utami Atmoko SS, Pamungkas J *et al*: **Parentage-based pedigree reconstruction reveals female matrilineal clusters and male-biased dispersal in nongregarious Asian great apes, the Bornean orang-utans (Pongo pygmaeus)**. *Molecular Ecology* 2012, **21**(13):3352-3362.
5. Nater A: **Processes underlying genetic differentiation and speciation in orangutans (Pongo spp.)**. *Dissertation.* University of Zurich; 2012.
6. Nietlisbach P, Nater A, Greminger M, Arora N, Krützen M: **A multiplex-system to target 16 male-specific and 15 autosomal genetic markers for orang-utans (genus: Pongo)**. *Conservation Genetics Resources* 2010, **2**(1):153-158.
7. Arora N, Nater A, van Schaik CP, Willems EP, van Noordwijk MA, Goossens B, Morf N, Bastian M, Knott C, Morrogh-Bernard H *et al*: **Effects of Pleistocene glaciations and rivers on the population structure of Bornean orangutans (Pongo pygmaeus)**. *Proceedings of the National Academy of Sciences* 2010, **107**(50):21376-21381.
8. Pritchard JK, Stephens M, Donnelly P: **Inference of population structure using multilocus genotype data**. *Genetics* 2000, **155**(2):945-959.
9. Locke DP, Hillier LW, Warren WC, Worley KC, Nazareth LV, Muzny DM, Yang S-P, Wang Z, Chinwalla AT, Minx P *et al*: **Comparative and demographic analysis of orang-utan genomes**. *Nature* 2011, **469**(7331):529-533.

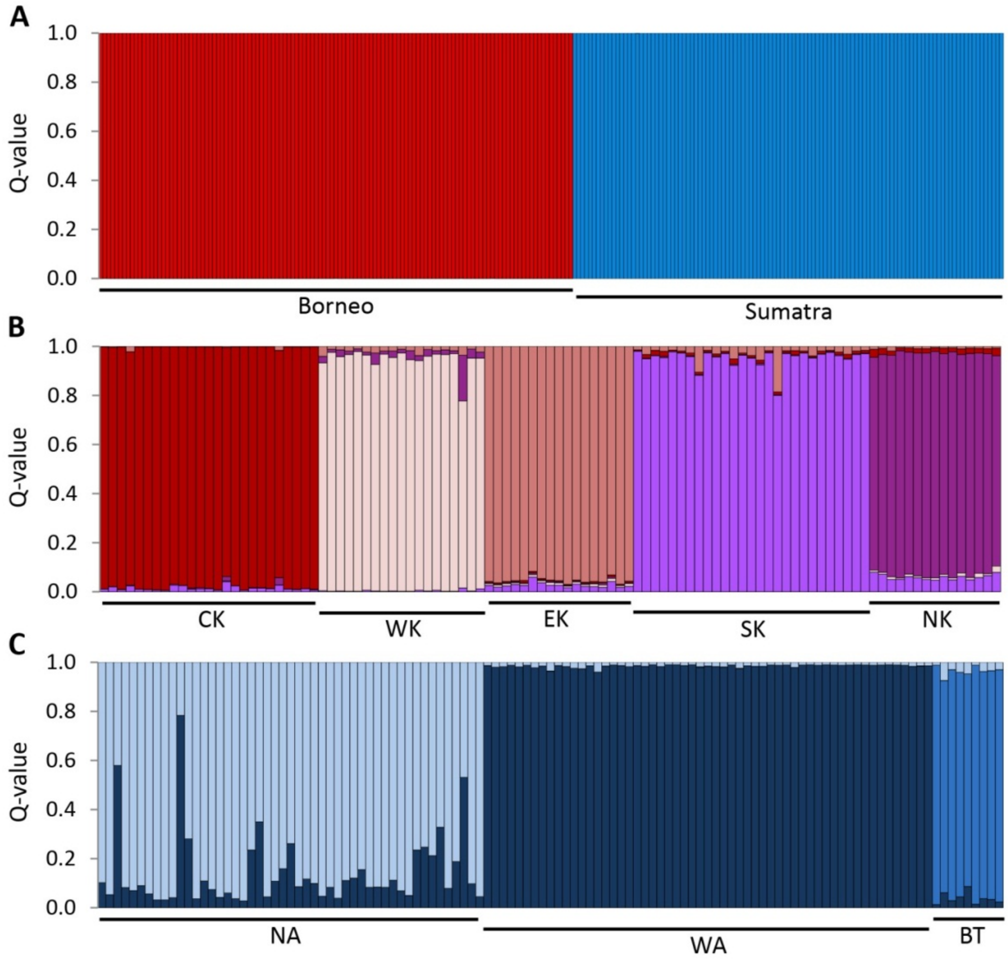

Figure S1. **STRUCTURE analyses to identify the population origin of study individuals.** The analyses were based on 27 microsatellite markers. The membership coefficients Q are average values over ten iterations with the same model parameters (admixture model with burn-in of $3x10^5$ followed by $3x10^6$ MCMC steps). Each bar represents a single individual. **(A)** Bornean and Sumatran individuals separate at the highest hierarchical level. **(B)** Within Borneo (n=104) we observe five clusters (most likely number of clusters as inferred by Arora *et al*. [7] and Nater [5]). Study individuals (n=16) were sampled from the South Kinabatangan cluster. CK: Central Kalimantan (n=25), WK: West Kalimantan (n=20), EK: East Kalimantan (n=17), SK: South Kinabatangan (n=25), NK: North Kinabatangan (n=17). **(C)** Within Sumatra (n=115) we observe three clusters (most likely number of clusters as inferred by Nater *et al*. [1] and Nater *et al*. [5]). Study individuals (n=15) were sampled from the West Alas population. NA: North Aceh and Langkat (n=49), WA: West Alas (n=57), BT: Batang Toru (n=9).

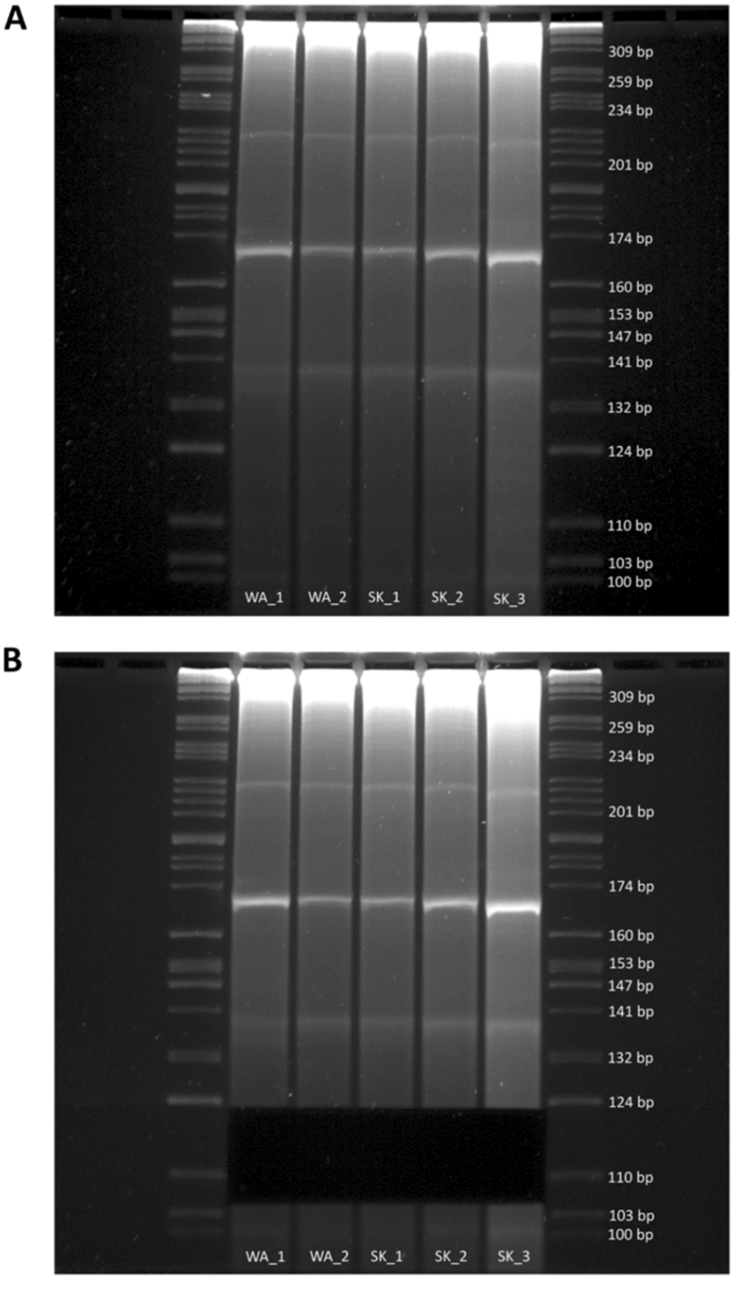

Figure S2. Example image of high precision excision of target fragments from Spreadex gel. *Hae*III digested genomic DNA was separated on high-resolution Spreadex EL400 Wide Mini S-2x13 gels with M3 size marker. Bands at e.g. 101 bp, 136 bp, and 170 bp represent repetitive elements as predicted by the *in-silico Hae*III digest of the orangutan reference genome (see Figure 2). **(A)** Image of a gel prior to fragment excision. **(B)** Image of the same gel after excision of DNA fragments in the target size range 104-123 bp.

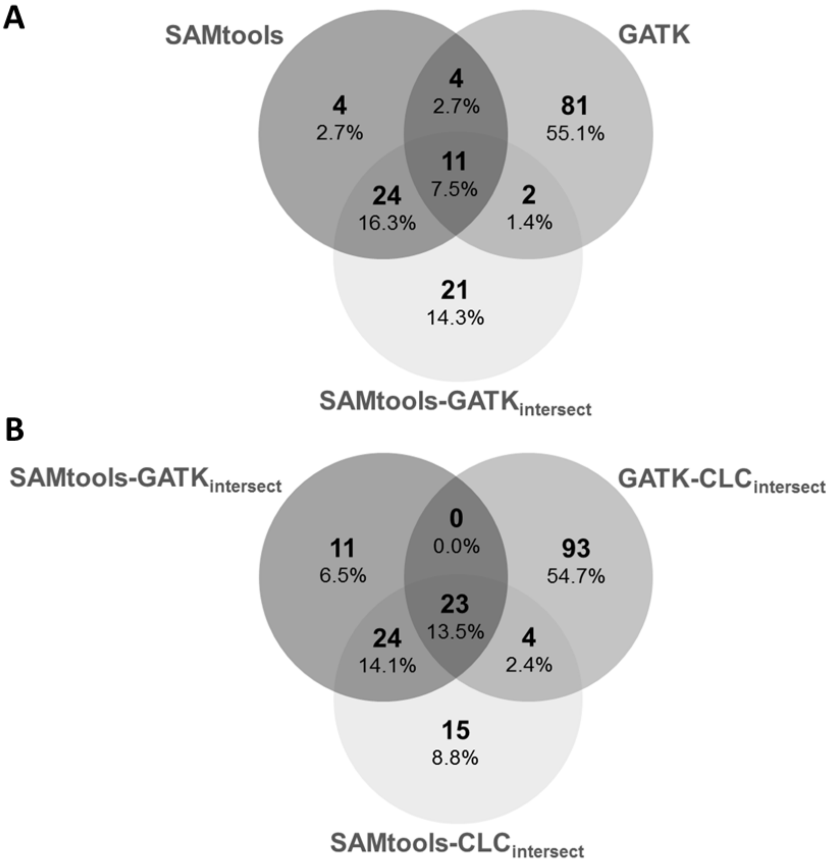

Figure S3. Overlap of outlier regions among SNP datasets in genome-wide scans for positive selection. For all SNP datasets we performed sliding-window analyses (100 kb window, 25 kb step size) of the absolute allele-frequency differential (*D*) between the South Kinabatangan and West Alas population. All windows with an average window $D > 0.95$ were considered as outliers, i.e. candidate regions for selective sweeps. **(A)** Comparison of SAMtools and GATK with their intersect dataset. **(B)** Comparison of the three intersect datasets.
